# Prezygotic Barriers to Hybridization in Marine Broadcast Spawners: Reproductive Timing and Mating System Variation

**DOI:** 10.1371/journal.pone.0035978

**Published:** 2012-04-26

**Authors:** Carla A. Monteiro, Ester A. Serrão, Gareth A. Pearson

**Affiliations:** CCMAR - CIMAR, University of Algarve, Gambelas, Faro, Portugal; University of Arkansas, United States of America

## Abstract

Sympatric assemblages of congeners with incomplete reproductive barriers offer the opportunity to study the roles that ecological and non-ecological factors play in reproductive isolation. While interspecific asynchrony in gamete release and gametic incompatibility are known prezygotic barriers to hybridization, the role of mating system variation has been emphasized in plants. Reproductive isolation between the sibling brown algal species *Fucus spiralis*, *Fucus guiryi* (selfing hermaphrodite) and *Fucus vesiculosus* (dioecious) was studied because they form hybrids in parapatry in the rocky intertidal zone, maintain species integrity over a broad geographic range, and have contrasting mating systems. We compared reproductive synchrony (spawning overlap) between the three species at several temporal scales (yearly/seasonal, semilunar/tidal, and hourly during single tides). Interspecific patterns of egg release were coincident at seasonal (single peak in spring to early summer) to semilunar timescales. Synthesis of available data indicated that spawning is controlled by semidiurnal tidal and daily light-dark cues, and not directly by semilunar cycles. Importantly, interspecific shifts in timing detected at the hourly scale during single tides were consistent with a partial ecological prezygotic hybridization barrier. The species displayed patterns of gamete release consistent with a power law distribution, indicating a high degree of reproductive synchrony, while the hypothesis of weaker selective constraints for synchrony in selfing versus outcrossing species was supported by observed spawning in hermaphrodites over a broader range of tidal phase than in outcrossers. Synchronous gamete release is critical to the success of external fertilization, while high-energy intertidal environments may offer only limited windows of reproductive opportunity. Within these windows, however, subtle variations in reproductive timing have evolved with the potential to form ecological barriers to hybridization.

## Introduction

Speciation occurs by the evolution of reproductive barriers that ultimately prevent genetic exchange between previously interbreeding populations [1]. Prezygotic isolating mechanisms range from ecological barriers to gametic incompatibility, while postzygotic isolation may arise through hybrid inviability, sterility, or reduced hybrid fitness. Ecological barriers are commonly the first to arise, and are more important than intrinsic postzygotic factors (reviewed for plants by [2]). In the sea, where reproduction by external fertilization is common, it may be more difficult for extrinsic ecological barriers to arise, resulting in more opportunities for hybridization [3]. Asynchronous gamete release between related taxa, and/or gametic incompatibility are considered the major prezygotic barriers to hybridization between externally fertilizing sympatric animal species [4]. In contrast to terrestrial plants, where mating system (particularly selfing) can maintain almost complete reproductive isolation between sympatric species in the presence of gene flow [5], outcrossing, albeit at varying rates, appears to dominate in hermaphroditic broadcast spawners [6].

The intertidal zone imposes unique constraints on external fertilization synchrony because during tidal emersion efficient gamete mixing is prevented and abiotic stresses can be intense, while during immersion hydrodynamic conditions may result in rapid gamete dilution and/or shear stresses on gametes that may severely reduce fertilization success [7], [8]. Despite this, broadcast spawning is a common reproductive mode in diverse marine intertidal taxa, from invertebrates to macroalgae [9]–[15].

Synchronous gamete release is critical to reproductive success in broadcast spawners, but the cyclical fluctuation of environmental conditions arising from interacting cycles at diurnal, tidal, semilunar and seasonal temporal scales suggests that external fertilization in intertidal species may be constrained within narrow windows of opportunity. We predict that selective pressures to restrict gamete release within these temporal windows will be strong. Indeed, some intertidal taxa possess sophisticated mechanisms to sense the environment and restrict gamete release, particularly to periods of low environmental water motion (e.g., in fucoid algae, [16]–[19]), maximising the probability of gamete encounters. However, some species seen to be stimulated to spawn by storms (e.g. *Patella* sp.; [20]).

A major question in the ecology of broadcast spawning taxa is whether patterns of gamete release synchrony at any particular temporal scale may act as a pre-zygotic barrier, a process identified at very fine scale in corals (see review by [21] for details). When hybrids display reduced fitness relative to parental taxa, selective constraints for maximizing reproductive success should be counterbalanced by selection to reinforce pre-zygotic barriers to hybridization. This may be achieved by interspecific variation in spawning time [4], such that congeneric gamete encounters are minimized. A second pre-zygotic mechanism may be mating system variation, particularly self-fertilization within hermaphroditic species, which is expected to dramatically reduce hybridization where individuals release male and female gametes in close proximity with minimal sperm limitation. An alternative to pre-zygotic barriers maintaining species coherence in the face of hybridization, is that hybrid fitness is conditional on the environment [22], and that hybrids are either favoured, or selectively neutral, under certain conditions or in particular microhabitats along the intense gradients of selection in the intertidal.

Species capable of coexistence despite hybridization and introgression remain one of the best and most challenging models to study the nature and role of reproductive barriers. This is the case of three sister species with different reproductive modes and mating systems; hermaphroditic *Fucus spiralis* and *Fucus guiryi*, in which selfing is the dominant form of reproduction throughout the studied range [23], [24], and dioecious, outcrossing *Fucus vesiculosus*. These species coexist as distinct entities [23], [25], [26] in the mid to high shore throughout most of their range in the eastern Atlantic [27]. Analyses of both multilocus genotypes [23] and variation in sexual phenotype [28] indicate that hybridization with introgression occurs, and therefore that hybrids are reproductively successful.

Investment into male function is an order of magnitude lower in *F. spiralis* compared with *F. vesiculosus*, suggesting that sperm limitation is not an important factor in the hermaphrodite [28]. If synchrony is selectively maintained primarily to ensure gamete encounters, then selfing hermaphrodites should display a relaxed synchrony relative to dioecious, obligately outcrossing species.

In this study we examined reproductive periodicity from seasonal to hourly timescales for the hermaphroditic fucoids *F. spiralis* and *F. guiryi*, and dioecious *F. vesiculosus*. This allowed us to test two predictions: 1) Reproductive synchrony is more important and therefore under stronger selective constraint in obligate outcrossers (*F. vesiculosus*) than in selfing species (*F. spiralis* and *F. guiryi*); 2) The timing of gamete release should vary between potentially hybridizing species in order to reinforce prezygotic (ecological) barriers to fertilization. Our data support both predictions, and also emphasize the ecological, developmental, and/or physiological constraints that operate to restrict reproduction to narrow temporal windows of opportunity in the intertidal zone.

## Materials and Methods

### Ethics Statement

No specific permits were required for the described field studies. No specific permissions were required for these locations and activities. The location is not privately-owned or protected in any way. The field studies did not involve endangered or protected species.

### Model organisms

The genus *Fucus* develops specialized reproductive tissue called receptacles on some apical tips of the thallus, and inside the receptacles are numerous spherical conceptacles. Depending on the species, sperm and egg develop inside the same conceptacle (i.e., hermaphroditic species, such as *F. spiralis* and *F. guiryi*); or in separate conceptacles from male and female individuals (i.e., dioecious species, as in *F. vesiculosus*). The three model species can be distinguished morphologically as described in Zardi et al. [26]. In all species, sperm and eggs develop inside gametangia called antheridia and oogonia, respectively, and both are released unfertilized (see review [18] for details). Fertilization is external and *Fucus* eggs are large (ca. 70 µm diameter), negatively buoyant and settle immediately as they are released. Sperm are negatively phototactic. Empirical studies [29]–[31], as well as local patterns of genetic structure [23], [24], [32], [33] all indicate that gamete and zygote dispersal is highly restricted. Eggs tend to fall immediately below the releasing alga [31] allowing, in settlement studies conducted in mono-specific patches, the assignment of egg origin to species with a high degree of confidence. The vertical distribution of these species on the shore is distinct although with overlapping zones [26], [34]; *F. spiralis* occurring mainly above *F. guiryi*, and these above the dioecious species *F. vesiculosus*. Hybrids between these species are uncommon and can be found mainly in the intertidal ranges where their parental species overlap [34]; they are fertile and appear as vigorous as their parental species [23], [28]. The restriction of hybrids to vertical contact zones is additional support for the low dispersal of gametes.

### Study site and sampling

**Figure 1:**
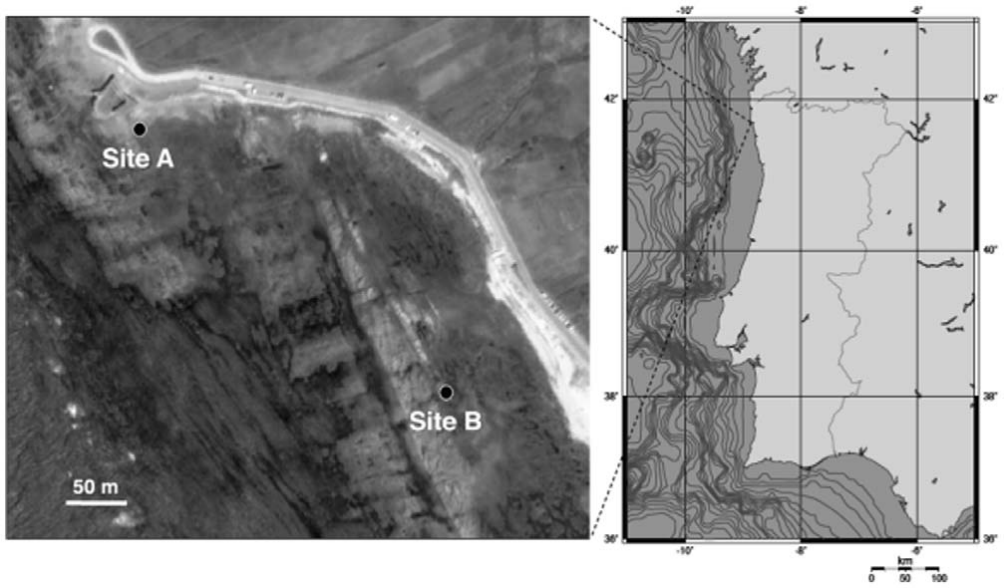
Map showing the location of the study site at Viana do Castelo, Portugal, and detailed view of the shore indicating the positions of the sampling sites A and B.

The study was conducted on a rocky shore at Viana do Castelo (Northern Portugal), at Praia Norte (Fig. 1). This location is protected from severe wave action by offshore rocky bluffs. The region is the southernmost limit for the sympatric occurrence of *F. vesiculosus*, *F. spiralis* and *F. guiryi* in Europe, and hybridization is more frequent in this contact range [35], possibly as a consequence of lack of reinforcement in southern allopatric populations.

The distribution of these species continues to the south, but *F. vesiculosus* is confined to estuaries and sheltered coastal lagoons, while *F. spiralis* and *F. guiryi* continues to occur on the open coast where suitable rocky substrate is available.

Sampling at seasonal – semilunar timescales

### Periodicity of egg release

The periodicity of eggs settled was monitored daily from February 2002 to July 2003, in two sites for *F. vesiculosus* and one site for *F. spiralis* and *F. guiryi*. The eggs were collected on artificial substrates (each with 5.96 cm^−2^) with a complex surface to promote egg retention (substrate preparation, fixation and sampling were as described in [11]). Ten disks (or fifteen at sites where due to strong wave action disk loss appeared more likely) were fixed under the algal canopy in Site A and B, respectively. Disks were replaced daily during low tide and were returned to the laboratory where egg release was quantified under a dissecting microscope. Eggs of both species are morphologically similar; all eggs, which settled on disks, were assumed to be from the species that cover the disks, since *Fucus* eggs tend to fall within less than 0.5 m of the releasing alga [31] and at each site the nearest individuals of any other *Fucus* species were more than 5 m away.

### Periodicity of receptacle maturation

In order to compare the temporal variability of gamete maturation in both species, total and mature oogonia were quantified every two days during 2002 and once a week during 2003. In five haphazardly chosen individuals within each site and species, one receptacle was collected at low tide. The total number of oogonia and the mature oogonia were quantified under a microscope in 3 conceptacles of each receptacle. Mature oogonia were defined as those in which cleavage furrows were evident (as in [17]).

### Sampling at hourly – tidal timescales

The timing of egg release during the day was studied in June to July 2009, in natural populations of *F. vesiculosus*, *F. spiralis* and *F. guiryi*. Along the vertical direction, the distance between species/entities was approximately 10 m, and at each height female gamete release by 5 individuals (females only for *F. vesiculosus*) was monitored in each of two replicate sites separated by approximately 5 m. Nylon mesh bags (40 µm) were used to enclose 2–3 receptacles per individual. A mesh size of 40 µm was chosen in order to retain eggs, while allowing water to circulate as freely as possible. The bags were attached to individuals with plastic clips with neoprene seals to provide a full seal around the thalli. During neap tide days, eggs were sampled every 2 h between 6:00 and 20:00, with a final sample taken at 21:30, prior to darkness. Bags were replaced carefully to prevent any egg loss in the field and were immediately taken to the laboratory where eggs were counted under a dissecting microscope. The bags were collected underwater or out of water, according to the tidal levels at each sampling time. At maximum high tide it was not possible to sample *F. vesiculosus* and the results presented are for 2 h periods before and after the high tide.

### Data analysis

We tested the hypothesis that relaxed constraint on reproductive assurance due to selfing in *F. guiryi* has led to a reduction in reproductive synchrony, relative to the obligate outcrossing species *F. vesiculosus*. To do this, we used two-tailed *F*-tests (degrees of freedom are the number of observations-1 for each of the distributions) to compare the variance in the distribution of spawning times (defined as the time of the first high tide after sunrise) for spawning events of a particular threshold magnitude (e.g. ≥10% or ≥20% of the maximum value). These data were obtained from the complete seasonal – semilunar settlement dataset from Feb 2002–Jul 2003.

The cumulative frequency distribution of egg release during neap tide cycles (2 h sampling intervals; see above) was plotted against ranks (i.e., Pareto or rank/frequency plots). For this, egg counts for each bag (n = 10 bags per 2 h sampling interval) were used to generate cumulative ranks, starting with the largest number of eggs released and adding sequentially the next largest observation until all observations have been summed. Linear regression lines were then fitted to such cumulated frequencies plotted in rank order and using logarithmic scales on both axes. Pairwise comparisons of linear regression slopes between species/entities fitted to double logarithmic plots were performed by standard techniques using dummy (or indicator) variables to compare linear models using the R statistical package and a custom script. In addition, the distributions were also compared using more robust non-parametric Kolmogorov-Smirnoff tests.

## Results

### Seasonal periodicity of reproductive output

**Figure 2:**
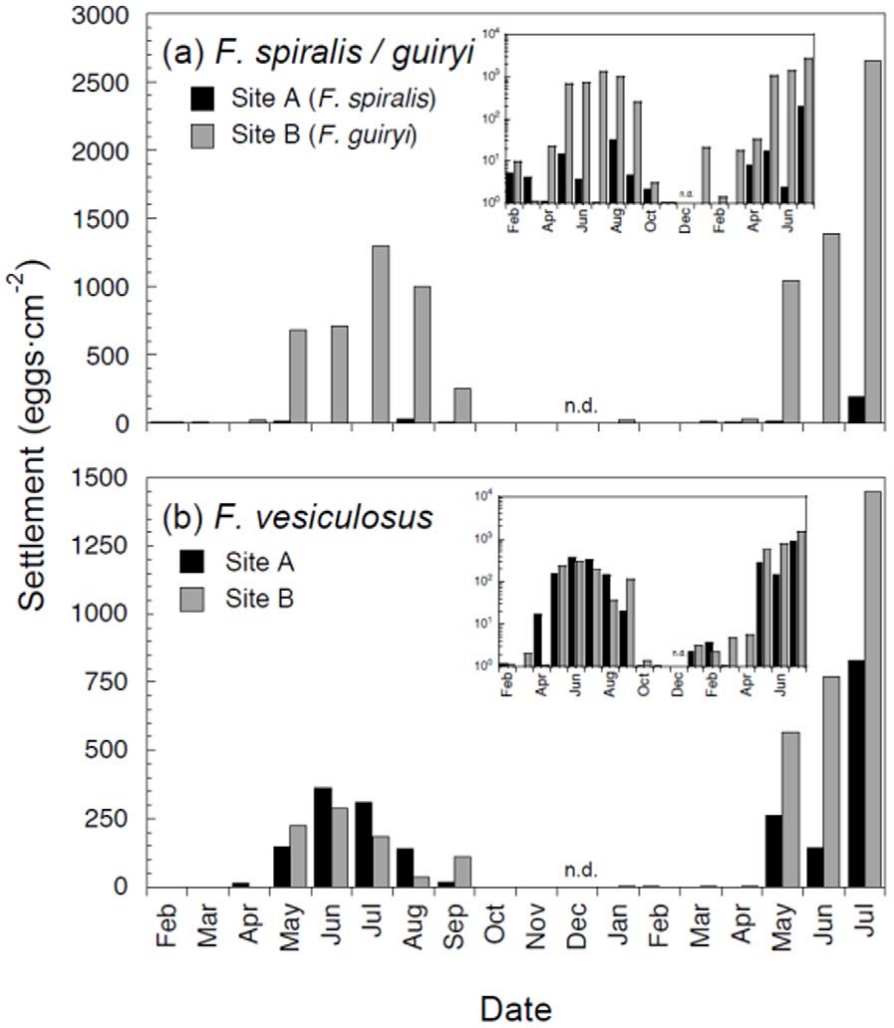
Monthly egg settlement from *Fucus spiralis* and *Fucus guiryi* (a) and *Fucus vesiculosus* (b) in site A (dark bars) and site B (grey bars) between February 2002 and July 2003. Periods when sampling was not carried out are represented by n.d. (no data). Insets show the data plotted on a Logarithmic scale.

Gamete release assessed by egg settlement in the field over 2 years was strongly seasonal in all study species (Figs. 2a and b). At a monthly temporal scale the patterns were indistinguishable between the three species. Although some egg settlement was detected throughout the year (see Figs. 2a and b insets plotted on Log scale), major settlement was detected only in May to September in 2002, and also only after May in 2003, until at least July 2003, when the survey was completed.

**Figure 3:**
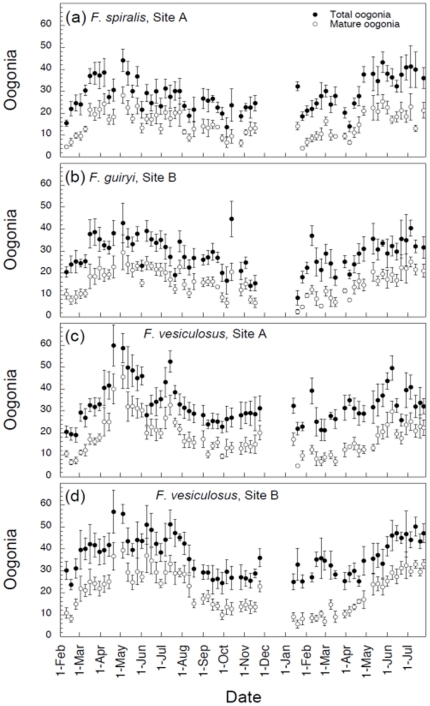
Total number of oogonia (closed symbols) and number of mature oogonia (open symbols) per conceptacle (n = 5 receptacles, ± SE) from *Fucus spiralis* (plot a), *Fucus guiryi* (plot b), and *Fucus vesiculosus* (plots c and d) between February 2002 and July 2003.

Both total and mature oogonia were observed throughout the year, peaking in April–May, immediately prior to the onset of major settlement events (Figs. 3a and b). During the main reproductive season there was a general decline in oogonia, although numbers were similar in periods when settlement was still observed (in August) and when settlement was near zero (October–November). Abundance of mature receptacles declined following the main reproductive period, coinciding with declining reproductive output (pers. obs.). However, the continued presence of mature oogonia following the end of the main reproductive period in October suggests that gamete release may not be directly related to the availability of gametangia.

Consistently lower settlement occurred in *F. spiralis* (site A), which had low density of reproductive individuals in the immediate vicinity of settlement disks. We included these data in Fig. 2a to illustrate that settled eggs originated from reproductive individuals immediately adjacent to sampling disks. Egg settlement quantification at the study site at various distances from the nearest females also consistently demonstrated low dispersal (Monteiro, Pearson, Serrão, unpubl. data). In addition to supporting the absence of interspecific contamination between sites monitored for each species, the ca. 2-fold lower settlement observed in *F. vesiculosus* compared with *F. guiryi* (site B, Fig. 2) may also be related to the low egg dispersal, since all individuals of hermaphroditic species produce eggs, compared with only half of dioecious *F. vesiculosus* (assuming equal sex-ratios); randomly-placed settlement substrates closer to males than females may therefore account for the reduced overall egg count for *F. vesiculosus*.

### Periodicity of gamete release on a daily scale within the semilunar cycle

**Figure 4:**
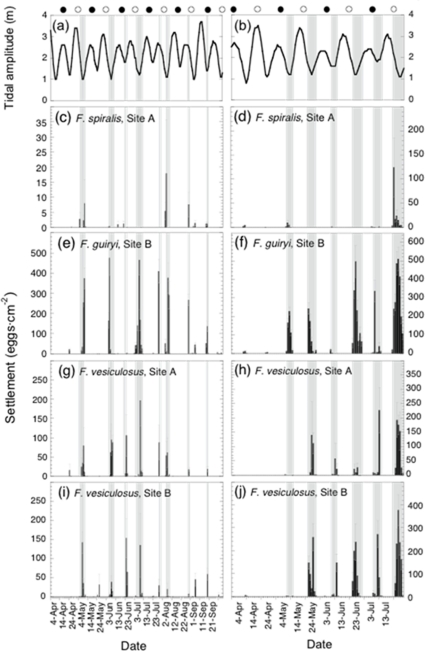
Daily egg settlement from *Fucus spiralis* (panels c and d), *Fucus guiryi* (panels e and f) and *Fucus vesiculosus* (panels g–j), between 1 April and 31 September 2002 (left hand panels) and 1 April and 31 July, 2003 (right hand panels). Values are means ± SE from n = 10 (Site A) or n = 15 (site B) sampling disks. Tidal amplitude (black line) and lunar phase for the sampling period are shown in a) and b); grey shading indicates the timing and duration of major settlement events from both species/sites.

On a finer daily scale during the semilunar cycle, gamete release was highly discrete, with peaks of release coinciding with neap (minimum amplitude) tides in both 2002 and 2003 (Fig. 4). In 2002, 9 major settlement events, and in 2003 a further 6 were recorded over a total of 311 days. In most cases, release occurred over 1–2 days, more rarely with an additional 1–2 days of low release before or after the main peak. In 2003, release events were somewhat broader in time (5–6 days, compared with 1–2 days in 2002) and peak settlement was higher than in 2002. Greater gamete release in 2003 may have contributed to the temporal broadening of release events as a result of sampling efficiency (i.e., increased detection efficiency of larger release events may have contributed to apparent peak broadening). Overall, the data show that the large majority of days on which release occurred were shared between species, and reveal no temporal barriers to interspecific gamete encounters in the water column at a semilunar time scale.

**Figure 5:**
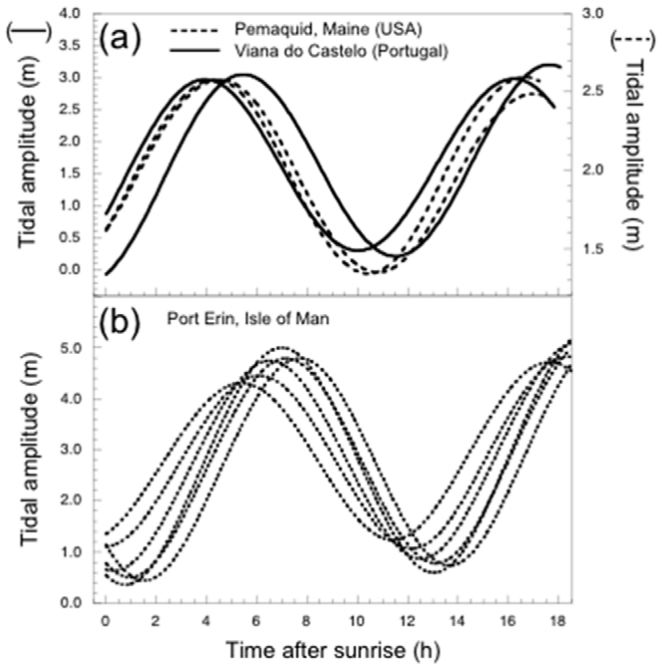
Relationship between tidal amplitude and time after sunrise on days with peak gamete release from a) *Fucus vesiculosus*, and b) *Fucus ceranoides*. Data are from Pemaquid, Maine, USA (Oct 8 and Nov 8, 1999; from Berndt et al. [9]), Viana do Castelo, Portugal (Jun 23, Jul 22, 2003; this study), and Port Erin, Isle of Man (Jul 17, Aug 1–3, 1989, and Jul 22, Aug 4, 1990; from Brawley [10]).

We compared the daily tidal phase on days of major gamete release in this study with those of Berndt et al. [9] for *F. vesiculosus* on western Atlantic shores in Maine, USA, because these authors observed release during spring tides, while we observed release exclusively during neap tides. However, the tidal phase (i.e., the timing of the low and high tides during the day) when gamete release occurred was very similar in both studies (Fig. 5a). Gamete release in *F. vesiculosus*, both in our study and in Berndt et al. [9], happens mainly on days when the first diurnal high tide occurs in the late morning. In Figure 5b we added tidal data from Brawley [10] for days with maximum gamete release in the estuarine dioecious fucoid, *F. ceranoides*. In this case peak gamete release appears shifted forwards by about 2 h relative to *F. vesiculosus*.

### Interspecific comparison of synchrony on a daily scale within the semilunar cycle

**Figure 6:**
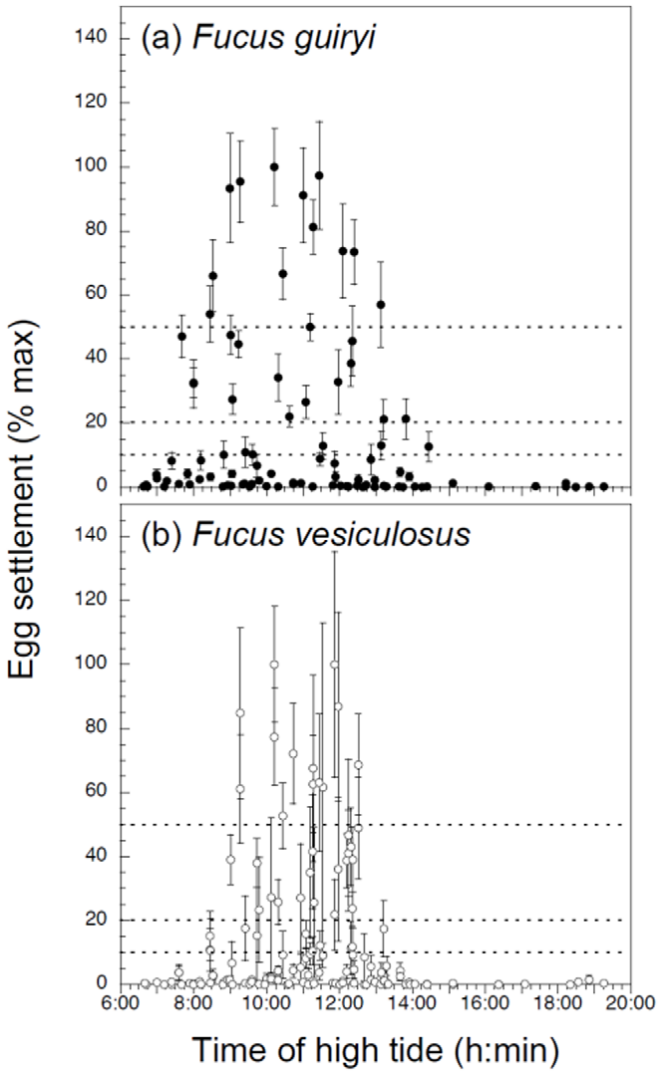
Relationship between the timing of high tide and egg settlement (shown as a percentage of the maximum) in a) *Fucus guiryi*, and b) *Fucus vesiculosus* at site A and B. Data are daily samples taken between February 2002 and July 2003 (n = 10 and 15 disks at site A and B, respectively, ± SE).

To compare interspecific reproductive synchrony within semilunar cycles, we used the daily egg settlement data from 2002–2003 to compare the variance in the timing of settlement events above certain threshold values. The data were normalized as a percentage of the maximum value observed, and plotted against the time of the first daylight high tide (i.e., phase in the semilunar cycle) on the day of release (Fig. 6). Gamete release from hermaphroditic *F. guiryi* had a higher variance (i.e., was less synchronous) with respect to semilunar phase than that from *F. vesiculosus*. Mean settlement values ≥10% of the maximum, occurred on days when the maximum level of the day-time high tide (defined as the first high tide after sunrise) fell within the time interval 07:41–14:27 h in *F. guiryi* (a range >6 h), but were restricted to days with peak high tides within the time interval 08:27–13:13 h in *F. vesiculosus* (ranging ca. 4:46 h) (two-tailed F-test; F_(32, 41)_ = 2.083, P = 0.027). For settlement events of ≥20% of the maximum the values were 07:41–13:49 h in *F. guiryi* (06:08 h) and 09:01–12:31 h in *F. vesiculosus* (03:30 h) (two-tailed F-test; F_(26, 30)_ = 2.772, P = 0.008).

### Intraspecific timing and synchrony of gamete release: hourly scale during the tidal cycle

**Figure 7:**
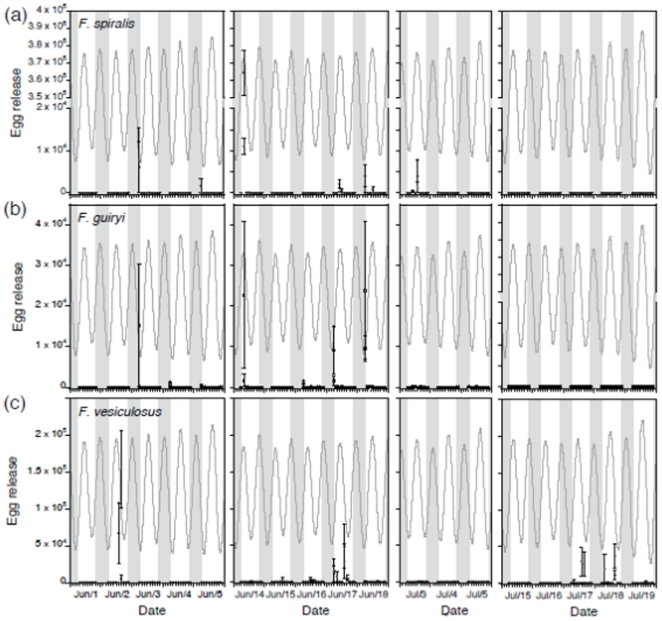
Egg release during 2 h sampling intervals (n = 5 ± SE) by a) *Fucus spiralis*, b), *Fucus guiryi*, and c) *Fucus vesiculosus* at two replicate sites/species (open and closed symbols) during four neap tide periods in June and July 2009. Grey lines are tidal amplitude and grey bars the dark periods during the daily cycle.

During the 4 neap tide periods studied, major egg release events (defined as >1000 eggs per bag in a 2 h sampling period) were observed on 8 days in *F. vesiculosus* (June 2–3, June 15–17, and July 17–19); 4 days in *F. guiryi* (June 3, 14, 17 and 18); and 6 days in *F. spiralis* (June 3, 5, 14, 17, 18 and July 3). While the three species showed co-occurring gamete release on several days, the largest events were shared mainly by the two hermaphrodites, *F. spiralis* and *F. guiryi*. (Figs. 7a and b), to the exclusion of *F. vesiculosus* (Fig. 7c). Moreover, the timing of gamete release within the tidal cycle was divergent between *F. vesiculosus* and the two hermaphrodite species. While *F. vesiculosus* released gametes almost exclusively immediately prior to, and especially following, the high tide (mid-morning to early afternoon), *F. spiralis* and *F. guiryi* released gametes consistently at the earliest sampling time of 06:00. Although we were unable to measure gamete release during peak high tide in *F. vesiculosus* due to the difficulty of sampling immersed individuals *in situ*, our data are consistent with this being the case, as reported by Berndt et al. [9] who observed the onset of release occurring prior to immersion. The precise timing of release in *F. spiralis* and *F. guiryi* remains uncertain, with both the dark to dawn transition, or release in the dark after the last sampling bags were attached (i.e., after 21:30 on the previous day) being possible.

**Figure 8:**
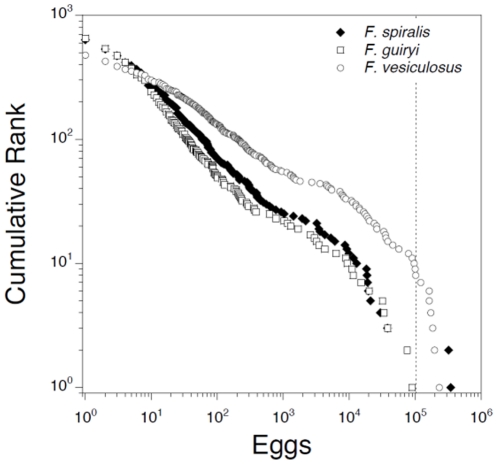
Pareto double Log_10_ plot of cumulative rank distribution of egg release. Data were collected during 2 h sampling intervals over four neap tide periods in June and July 2009 (see text and Fig. 7). Summary results of the regressions are 1) *Fucus spiralis*: Log(y) = 2.853−0.476*Log(x), R^2^ = 0.981; 2) *Fucus guiryi*: Log(y) = 2.767−0.488*Log(x), R^2^ = 0.970, and 3) *Fucus vesiculosus*: Log(y) = 2.812−0.345*Log(x), R^2^ = 0.987.

Double logarithmic plots of cumulative frequency against ranked egg release magnitude for 2 hourly intervals (Pareto plots) revealed power-law distributions for all three entities (*F. spiralis*, *F. guiryi* and *F. vesiculosus*; Fig. 8). The relationship deviated from linearity only at values of egg release >ca. 10^5^ eggs per sampling bag (2–3 receptacles), which probably approaches the upper limit of eggs contained in the sample receptacles. The regression fit to the *F. vesiculosus* data was improved (r^2^ = 0.987 *versus* 0.952) by removing values ≥10^5^, so this was chosen as a cut-off value for number of eggs sampled. The fit of the regressions were also high for hermaphrodite species (r^2^ = 0.9813 and 0.9701 for *F. guiryi* and *F. spiralis*, respectively), and were highly significant in all cases (*P*<0.0001). Pairwise comparisons of the regression slopes showed no significant difference between the two hermaphrodite species (*P* = 0.208), but that both were significantly different from *F. vesiculosus* (*P*<0.0001 in both cases). The same was found using Kolmogorov-Smirnoff tests; the distributions of egg release data did not differ between the hermaphroditic species (*F. spiralis* versus *F. guiryi*, p = 0.800) but differed between these and the dioecious *F. vesiculosus* (*F. guiryi* versus *F. vesiculosus*, p = 0.000; *F. spiralis* versus *F. vesiculosus*, p = 0.000). It follows that the exponent or scaling factor, *α*, for the power law relationships calculated from the regression slopes varied little between the two hermaphrodite species (0.4760 and 0.4883 for the *F. spiralis* and *F. guiryi*, respectively), but was lower for *F. vesiculosus* (0.3454).

## Discussion

This study provides one of the most comprehensive descriptions to date of reproductive timing (egg release) at annual to hourly scales in the marine environment. We had two main goals; firstly to investigate the potential for interspecific variation in spawning time to act as a prezygotic ecological barrier to hybridization between sympatric, externally-fertilizing congeners. Our data show that, while coincident at seasonal and semilunar scales, differences in the timing of gamete release during single tides are consistent with a partial ecological barrier to hybridization. Secondly, we addressed the hypothesis that selfing species are under reduced selective constraint for spawning synchrony relative to obligately outcrossing species. We discovered that while reproductive synchrony remains a feature in selfing hermaphroditic species, it is reduced relative to sister species with obligate outcrossing. Reduced synchrony was identified on daily time scales as increased variance of major spawning periods during the semilunar reproductive cycle, and from a relative excess of small spawning events during single favourable tides. Finally, we provide evidence from these data and previously published studies on both sides of the Atlantic [9], [10] that gamete release in natural fucoid populations is controlled by environmental cues arising from the interaction of tidal and diurnal cycles, rather than semilunar cycles.

Spawning patterns at seasonal to daily temporal scales were highly coincident for *F. vesiculosus*, *F. spiralis* and *F. guiryi*. Reproductive output, estimated as daily egg settlement, a close proxy for gamete release [10], [15], [19], or oogonial maturation, followed essentially the same temporal pattern for the two hermaphrodite species *F. spiralis* and *F. guiryi*, and for dioecious *F. vesiculosus*. Maximum reproductive output occurred from late spring to summer (May–Sep). We are not aware of other comparative long term spawning datasets for *Fucus* spp. or other intertidal organisms. The restriction of most gamete release to massive spawning events over a few days during the reproductive season is typical, and parallels reports of mass reproductive events in broadcast spawning corals or green algae in tropical reef systems (e.g., [36], [37], see also review by [21]).

Gamete release in all study species followed a≈14 day period, synchronized with neap tides during semilunar cycles. Synchronous spawning with the same periodicity has been reported for both dioecious and hermaphrodite *Fucus* species [10]–[12], [15], [38]. However, the timing within the semilunar cycle we observed (i.e., neap tide periods) is out of phase with data for *F. vesiculosus* from the eastern Atlantic (Maine coast, USA) [9], [38], as well as those for an estuarine fucoid, *F. ceranoides* in the Irish Sea, UK [10], both of which show peak gamete release during spring tides around full and new moon. This apparent conflict is resolved by considering tidal, rather than semilunar cycles; tidal and diurnal cycles are similar between Maine and the Irish Sea, where mid – late morning high tides occur near full and new moons, whereas in northern Portugal, similar timing of high tides occurs during the neap period (i.e., around quarter moons). These results strongly suggest that gamete release in intertidal fucoids at the daily scale is entrained by the interaction between semidiurnal cycles of high and low tides and daily light-dark cues, rather than by cues arising from semilunar (spring – neap tide) cycles (Fig. 5). Similarly, a model combining fitness components associated with environmental cycles of differing periodicity was shown to underlie the apparent semilunar spawning patterns of a puffer fish [39]. While our data are compelling, independent confirmation could be obtained by comparisons of spawning times in natural populations in the eastern Atlantic, subject to a north – south gradient in the timing of tides [40] or in areas with striking tidal phase shifts along short distances, versus the western Atlantic, where there are no such tidal gradients.

At hourly time scales, spawning was largely synchronous between hermaphroditic *F. spiralis* and *F. guiryi*, but asynchronous between hermaphroditic and dioecious species, at daily and particularly hourly time scales (Fig. 7). Major spawning events in hermaphrodites occurred mainly on days when spawning by *F. vesiculosus* was very low or did not occur. Temporal reproductive isolation is a potentially effective means of reinforcing reproductive isolation, and a key ecological factor in sympatric speciation. Temporal isolation in sympatry has been considered unlikely [41], although empirical data from conspecific broadcast spawning algae and corals [4], [37], [42], as well as theoretical models [43] indicate that it does occur, with important evolutionary consequences for reproductive isolation and sympatric speciation in natural communities. These changes in synchrony and timing of reproduction in *Fucus* have evolved recently, since we now know that *F. spiralis* and *F. guiryi* most likely arose during the Pleistocene glacial cycles, and that their evolution coincided with a switch in reproductive mode from the ancestral dioecious state shared with *F. vesiculosus* [44]. Our data for *F. vesiculosus* broadly agree with those of Berndt et al. [9] in suggesting that major spawning events occur during daytime high tide immersion, since we observed that release began prior to, and continued after, the high tide (see also [12] for details). In contrast, major spawning events in hermaphrodite species were observed by the first sampling interval (06:00, around dawn), implying either that spawning occurred during the light to dark transition, during the night (the last sampling bags were placed at 21:30), or at the dark to light transition at dawn [37]. In this respect it is interesting to consider that a more distantly related member of the family Fucaceae (*Silvetia compressa*) releases gametes in the laboratory on a light to dark transition after a period under favourable conditions in the light ([16], reviewed by [18]), and can release gametes at low tide in the field [45]. Whatever the exact timing in hermaphrodite species, the interspecific shift in spawning of congeneric hermaphroditic and dioecious species observed here is consistent with selection favouring ecological reproductive isolation, since in congeneric corals a shift in spawning of only 2 h was inferred to reinforce reproductive isolation [4].

Two hypothetical selective mechanisms could have originated the temporal reproductive isolation between the dioecious (*F. vesiculosus*) and hermaphroditic (*F. spiralis* and *F. guiryi*) lineages, sympatric speciation or reinforcement against hybridization. Before the split of the hermaphroditic and dioecious lineages [44] mutations leading to spawning timing difference could have created the reproductive isolation that originated, or contributed to, speciation. Alternatively, after lineage split, reinforcement might have occurred if hybrids were less fit than their parental species along each specific vertical zone, in agreement with the observations that these species vertical distribution is correlated with different stress resilience [26] and that hybrids are found mainly in the contact zones along the vertical zonation [34]. In such a scenario, populations that remained non-introgressed due to different spawning times would have been positively selected.

The species *F. spiralis* and *F. guiryi* were only recently detected as distinct genetic entities with limited gene flow [26], [44]. We found no evidence for ecological prezygotic barriers to gene flow between these species, suggesting that either mating system is a sufficient hybridization barrier in these compatible and selfing [24]. entities, or that other pre- or postzygotic barriers exist. Mating system variation can reinforce speciation processes, e.g. a shift from outcrossing to selfing resulted in almost complete reproductive isolation between potentially hybridizing plant species [5]. However, while flowering plants rely on pollen vectors (e.g., insects) for cross-fertilization, or have developed effective selfing mechanisms like cleistogamous (non-opening) flowers, it is less clear how effective mating system variation can be in broadcast spawning marine external fertilizers. Temporal differences in spawning times occur also in sympatric and inter-fertile marine invertebrates, and may play a role in prezygotic reproductive isolation, reinforcing some degree of gametic incompatibility (e.g., [46]). Since syngamy occurs shortly after spawning, selfing rates presumably depend on the spatial proximity of the eggs and sperm released from the same individual. Eggs from different fucoid species share the same pheromonal sperm attractant [47], and dioecious male conceptacles produce an order of magnitude more sperm than hermaphrodites [28]. Therefore, as a hybridization barrier in mixed stands of broadcast spawning congeneric species, mating system alone is expected to be an imperfect isolating mechanism.

Theoretically, the selection pressure to maintain spawning synchrony in dioecious (i.e., obligately outcrossing) broadcast spawning species should be stronger than for self-fertilizing hermaphrodites. Separate male and female individuals are selectively constrained to ensure reproductive success by spawning into the water column at the same time, while individuals of selfing hermaphrodites can assure reproductive success by releasing male and female gametangia from the same reproductive tissue (conceptacles), and are not therefore constrained in the same way. Our finding of larger temporal variance in spawning time during semilunar cycles in *F. spiralis* and *F. guiryi* than in *F. vesiculosus* supports the hypothesis of a reduced constraint on synchrony in hermaphroditic species. Despite this, at a smaller temporal scale, power law-like distributions of spawning intensity during reproductive periods (neap tide cycles) were observed in all three taxa/genetic entities, indicating very few large release events and many small events. This indicates that, for both dioecious and hermaphroditic species, reproduction (and therefore recruitment, as the two processes are closely linked in fucoids) depend for their success on very limited temporal “windows of opportunity". During these rare events of synchronous spawning, the predominant form of crossing may still be between neighbouring individuals due to the limited gamete dispersal [31], which may explain high levels of biparental inbreeding observed in several *F. vesiculosus* populations [24]. Although our data support a reduced constraint on synchrony in hermaphroditic species at semilunar scales, at smaller, hourly, scales within peak release days, the opposite trend is observed, possibly resulting from their use of different environmental triggers for circadian release timing. The shallower slope (significantly lower α) of the power law relationship in dioecious *F. vesiculosus* (i.e., greater “evenness"), indicates less synchrony at hourly scales during the neap tide release periods. A possible reason for this contrast could be use of different cues for gamete release timing within circadian scales. Spawning in *F. vesiculosus* was observed to coincide with immersion at high tide, a more extended interval including the immediately prior and following emersion periods. In contrast, the shift to low tide (emersed) release in *F. spiralis* and *F. guiryi*, possibly in response to putative dark/light shift signals, appears to result in more synchronous release at this smaller temporal scale.

On a semi-lunar scale, our data support the prediction that constraints on reproductive synchrony are reduced in selfing species in the intertidal. However, the broadly coincident semilunar spawning patterns, as well the power-law relationships for egg release, showing high synchrony for all entities independent of mating system, both indicate that any synchrony differences are quite small. Why is reproductive synchrony maintained in hermaphrodite species at all? Possible reasons include evolutionary contingency, given the very recent divergence of the two species [44], [47], maintenance of adaptive potential by some degree of continued outcrossing [48], [49], and/or ecological factors unrelated to reproduction directly. If *F. spiralis* and *F. guiryi* arose recently from a dioecious ancestral lineage [44], then synchrony may simply be a retained ancestral trait. Populations of *F. spiralis* and *F. guiryi* show evidence of high levels of inbreeding [23],[24], which may be favoured in marginal habitats (such as the upper intertidal shoreline where *F. spiralis* and *F. guiryi* occur), and where maintaining locally adaptive gene combinations and/or purging of deleterious alleles may be particularly advantageous (reviewed by [50]).. Nevertheless, the benefits of occasional outcrossing may be maintained via continued selection for reproductive synchrony. A third possible reason may relate to other life-history traits such as limiting dispersal to favourable potential habitats. Since in fucoids zygotes settle and develop immediately post-fertilization, the timing of spawning may also be selected for optimal recruit survival in the steep vertical abiotic gradients of the intertidal zone, as suggested earlier [18]. Releasing gametes at low tide by these hermaphroditic taxa might favour retention of propagules at their higher intertidal range as the upwards moving tide washes pre-released gametes onto local safe micro-sites. Thus gamete release at slack high tide [9], or at low tide in selfing species, besides maximising fertilization success, might simultaneously limit zygote wastage from dispersal out of their narrow favourable intertidal range.

Fucoid algae are a fascinating system in which to address, from an ecological viewpoint, some of the problems associated with maintaining reproductive barriers and more broadly with speciation/hybridization processes, in broadcast spawning marine species. The timing of reproduction in the intertidal zone may be particularly constrained for broadcast spawners, with very limited temporal windows for reproductive assurance imposed by immersion-emersion cycles and the dilution effects of surf zone hydrodynamism [51]. Therefore species with incomplete reproductive isolation must balance reproductive assurance (e.g., fertilization success under optimal hydrodynamic conditions) while minimizing hybridization with sympatric congeners. The species studied here have incomplete reproductive isolation, and low proportions of hybrids are commonly observed, mainly at contact zones [23], [25], [28], [34], but are nevertheless maintained as genetically distinct entities in sympatry.

While the seasonal, tidal and diurnal cues entraining gamete release are shared by sister taxa, a temporal shift in reproductive timing within single tides constitutes an ecological barrier to gene flow between the selfing hermaphrodite, *F. spiralis* and *F. guiryi* and dioecious *F. vesiculosus*. Mating system variation plays two important roles; while dioecious species require high tide immersion to mix gametes and a high degree of reproductive synchrony, selfing hermaphrodites are less constrained, possibly including reproduction during emersion at low tide [52]. The mechanism(s) preventing gene flow between the two hermaphroditic species *F. spiralis* and *F. guiryi* remains to be seen, but subtle differences in reproductive timing [4], gamete release during low tide emersion, and selfing [5] may, separately or in concert, be sufficient prezygotic barriers to maintain them as distinct genetic entities in sympatry.
